# Structural analysis of physical gel networks using graph neural networks

**DOI:** 10.1140/epje/s10189-024-00469-w

**Published:** 2025-01-13

**Authors:** Matthias Gimperlein, Felix Dominsky, Michael Schmiedeberg

**Affiliations:** 1 Institut für Theoretische Physik 1, Friedrich-Alexander-Universität Erlangen-Nürnberg, Erlangen, 91058 Bavaria Germany; 2 Max-Planck Institut für Physik, Garching, 85748 Bavaria Germany

## Abstract

**Abstract:**

We employ graph neural networks (GNN) to analyse and classify physical gel networks obtained from Brownian dynamics simulations of particles with competing attractive and repulsive interactions. Conventionally such gels are characterized by their position in a state diagram spanned by the packing fraction and the strength of the attraction. Gel networks at different regions of such a state diagram are qualitatively different although structural differences are subtile while dynamical properties are more pronounced. However, using graph classification the GNN is capable of positioning complete or partial snapshots of such gel networks at the correct position in the state diagram based on purely structural input. Furthermore, we demonstrate that not only supervised learning but also unsupervised learning can be used successfully. Therefore, the small structural differences are sufficient to classify the gel networks. Even the trend of data from experiments with different salt concentrations is classified correctly if the GNN was only trained with simulation data. Finally, GNNs are used to compute backbones of gel networks. As the node features used in the GNN are computed in linear time $\mathcal {O}(N)$, the use of GNN significantly accelerates the computation of reduced networks on a particle level.

**Graphic abstract:**

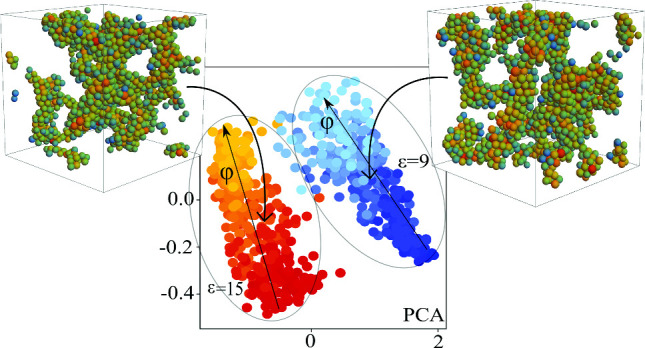

## Introduction

Gelation is an extensively studied phenomenon, which takes place in various physical many particle systems, such as colloid-polymer mixtures. The competition of hard-core-like repulsions, longer-ranged screened Coulomb repulsion [1, 2] and shorter-ranged effective depletion attraction [3–5] between the colloids close to contact can lead to the onset of a rich heterogeneous phase behaviour during gelation. At low packing fractions far above the binodal line dilute gel networks consisting of long, thin strands are found [6–10], while at larger packing fractions clumpy gels are observed. Further possible structures are homogeneous fluids below the phase separating line or clumpy fluids [11] very close to the phase separating line. Gelation is further characterized by a slowdown of dynamical evolution in the system [12–18].

In a recent work, we studied global and local structure formation and the differences between dilute physical gel networks and clumpy gels [19]. We found that at intermediate packing fractions a continuous crossover between the two gel types occurs [20]. On a first glance the structural differences are hidden in the internal structure of the gel network. They can be extracted from so-called reduced networks. These are minimal connecting structures determined by removing all particles from the network which are not crucial for the connectivity. The exact algorithm was introduced and employed in our recent works [19, 20]. Note that the structural differences at the crossover are small, but we have shown that the relaxation process close to the crossover changes [20]. Therefore, there are indeed qualitatively different types of gels if the dynamics is considered. However, it remains an open question whether the structural differences are sufficient for a classification.

Due to the large number of particles, the analysis of gel networks is computationally expensive. In this paper, we explore possibilities to accelerate the structural analysis and find hidden similarities among structures of the same type using a machine learning approach. We encode gel networks as graphs, where nodes are identified with particles and edges with interacting particles close to contact. These graphs are then used as input for graph neural networks (GNN) to analyse and classify the corresponding gel networks. GNNs are an extension of conventional machine learning algorithms for complex, non-Euclidean data structures [21–24]. They have been applied in the analysis of complex data with non-Euclidean neighbourhood relations, which can be translated to graph structures. Often the translation to graph structures is a challenging task. [25] Applications can be found in physics [26, 27], chemistry [28], traffic forecast [29] and social networks [30, 31]. Popular examples of such neural networks are graph convolutional networks (GCN) [32] or GraphSAGE [33]. In this article, we use graph isomorphism networks (GIN)[34] which are a special type of message passing graph neural networks [35]. This network type gathers and combines in each layer the node features of neighbouring nodes in the previous layer and uses them to compute a new output for each node. Therefore, the whole idea of message passing graph neural networks using adapted convolutional networks is inherently local. The number of layers in the network determines to how many layers of neighbouring nodes each node embedding has access. In this sense, a *k*-layer message passing GNN means that for each node the information gathered from the *k*-hop neighbour environment is available. Even though the approach is local, it is still possible to use it for the prediction of global properties of the graph.

In Sect. 2, we present our simulation procedure and the construction of graphs from simulated gel networks. Later in Sects. 3.1 and 3.2, we use GNNs for graph classification. Depending on the interaction strength and the packing fraction, we use these results to identify the correct position of the samples in the state diagram. Furthermore, the GNN is used to classify experimental data. Note that both a supervised learning approach and an unsupervised approach are presented. In Sect. 3.3, we use GNNs for a node classification and accelerate the computation of reduced gel networks. As the analytic computation of backbone structures on a particle level is computationally expensive, alternative approaches such as ArGSLab based on thinning binarized images have been developed [36]. The disadvantage of these approaches is that the result is on a pixel level, and not anymore on a particle level. We employ our GNN-approach to accelerate the determination of reduced networks. We find that GNNs are indeed capable of reconstructing big parts of the correct backbone structures. To get the best results for the predicted backbone structures, it is important to choose the classification threshold correctly. The prereduced network should then be postanalyzed with our analytical method. The big advantage of using neural networks for this task is that the time complexity of the node feature computation and the classification time complexity is of linear order $\mathcal {O}(N)$, while for the analytic computation algorithm it is at least of order $\mathcal {O}(N^2)$. In Sect. 4, we finally conclude and summarize our results. In Appendices A and B, we present our methods computing node features and using them for constructing graph neural networks. Furthermore, we present the final sets of hyperparameters and the detailed architectures of the GNNs.

## Methods

### Simulation procedure

The training and test datasets of gel networks are simulated using Brownian dynamics simulations as before in [19, 20]. To be specific, we consider a system of colloidal particles with a polydispersity of $5\%$. The particle sizes are drawn from a Gaussian distribution. The interaction is the sum of the two potentials $U_{SW}(r)$ and $U_{YK}(r)$ given by

$$\begin{aligned} U_{\text {YK}, ij}(r)&=C \left( \frac{2}{2+\kappa \sigma _{ij}}\right) ^2\left( \frac{\sigma _{ij}}{r}\right) \text {exp}[-\kappa (r-\sigma _{ij})],\\ U_{\text {SW},ij}(r)&={\left\{ \begin{array}{ll} -\frac{\epsilon }{2\alpha _{ij}} r+\frac{\epsilon (\sigma _{ij}-\alpha _{ij})}{2\alpha _{ij}} &  r<\sigma _{ij}+\alpha _{ij}\\ -\epsilon &  \sigma _{ij}+\alpha _{ij} \le r \\   &  r \le \sigma _{ij}{+}\delta _{ij}{-}\alpha _{ij}\\ \frac{\epsilon }{2\alpha _{ij}}r - \frac{\epsilon (\sigma _{ij}+\delta _{ij}+\alpha _{ij})}{2\alpha _{ij}} &  \sigma _{ij}{+}\delta _{ij}{-}\alpha _{ij}< r \\ &  r < \sigma _{ij}{+}\delta _{ij}{+}\alpha _{ij} \\ 0 &  \text {else}. \end{array}\right. } \end{aligned}$$

where $U_{SW}(r)$ is a modified attractive SW-potential and $U_{YK}(r)$ a long-range repulsive Yukawa tail [19, 20]. $\sigma _{ij}=r_i+r_j$, where $r_i$ is the radius of particle *i* is the minimal distance without overlap of two particles. $\epsilon$, $\delta _{ij}=0.03\sigma$ and $\alpha _{ij}=\frac{\delta _{ij}}{5}$ represent the depth of the SW-potential, its width and the flattening of the wells. The strength of the repulsive potential is modified by the screening length $\kappa ^{-1}$. The parameter *C* is chosen as 200$k_BT$. The force calculation is cut-off at a distance $\frac{r_{\text {Cut}}}{\sigma }=1+\frac{4}{\kappa }$ as in [37]. In summary, the whole system can be characterized by choosing a triplet of parameters $(\epsilon , \kappa , \varphi )$, where $\varphi =\frac{\pi }{6}\sigma ^3\frac{N}{L^3}$ is the packing fraction of the system with box size *L*. Note that in the following $\epsilon$ is used in units of $k_BT$ and $\kappa$ in units of $\sigma ^{-1}$. The trajectory of colloidal particles in a solvent is determined by numerically integrating the overdamped Langevin equation for particle *j*

$$\begin{aligned} \gamma \frac{{\text {d}}}{{{\text {d}}t}}\vec {r}_{j} = \vec {F}_{{\text {int} }} + \vec {F}_{{{\text {th}}}}. \end{aligned}$$

Here $\gamma$ is the friction constant, $\vec {F}_{\text {int}}$ models the effective force between colloidal particles as given by the pair interaction potential, and $\vec {F}_{\text {th}}$ denotes the random forces due to thermal fluctuations. It is $\left\langle \vec {F}_{\text {th}}\right\rangle =\vec {0}$ and $\left\langle F_{\text {th},i}(t)F_{\text {th},j}(t')\right\rangle =2\gamma k_\text {B} T\tilde{\delta }_{ij}\delta (t-t')$. Note that $\tilde{\delta }_{ij}$ here is the Kronecker-$\delta$ and not the interaction range $\delta _{ij}$ used above. The time steps in our simulations are $\Delta t=10^{-5} \tau _B$ with the Brownian time $\tau _B=\frac{\sigma ^2\gamma }{4k_\text {B} T}$. The calculation of forces is done using a combination of the Verlet-list algorithm and the linked-cell algorithm to reduce computation time [38]. For all considered packing fractions, boxes of size $20\sigma \times 20\sigma \times 20\sigma$ with periodic boundary conditions are used and filled with colloids until the considered packing fraction is reached.

### Data preparation

Training and test data are simulated for four different packing fractions ($\varphi =0.05, 0.10, 0.15, 0.20$). For each packing fraction, we consider two different interparticle attraction strengths ($\epsilon \in \{9, 15\}$). For each of these 8 different configurations from the phase diagram, 25 simulations are done. Twenty of these simulations are chosen as training datasets and 5 as test datasets. This splitting of the simulations guarantees that the test dataset cannot contain samples which are also part of the training dataset. For the evaluation, a third dataset is simulated containing 5 datasets for each density $0.05\le \varphi \le 0.20$ in steps of 0.01 and $\epsilon \in \{9, 15\}$. For the dilute systems ($\varphi \le 0.12$) gel network configurations are taken after $1000\tau _B$ simulation time, while for dense gels ($\varphi >0.12$) the configurations are already taken after $500\tau _B$. This choice is made to reduce computation time. It is justified as in dense systems heterogeneous structures form faster than in dilute systems.

For data preparation first a graph is constructed from the gel networks using the python module networkX [39] by identifying gel particles with nodes in the graph. An edge is inserted between a pair of nodes if the corresponding particles in the gel network touch each other. As touching criteria, we use the attractive distance between two particles. If this distance is smaller than the range of the attractive force between them, i.e. $r_{ij}<1.036\sigma$, the particles touch. The value $1.036\sigma$ is just $\sigma +\delta +\alpha$ for the chosen values of $\delta$ and $\alpha$. For the experimental procedure, we use a different touching criterion as the range of the attractive force is not known exactly. Therefore, we analyse radial distribution functions and decide to use $r_{ij}<2.2\mu m$ (particle diameter $\sigma _{exp} \approx 1.76 \mu m$ [18]) as a touching criterion. This is slightly larger than the value of the first minimum in g(r). The slightly larger distance is chosen as size variations and fluctuations usually are larger in experiments. Thermal fluctuations might lead to random splitting of bonds in single timesteps which influences the structure of the gel network. More details are explained in [20] for simulated gel networks.

Each node is assigned a set of 6 distinct node features (for more details, see Appendix A). The whole idea of graph neural network depends on the message passing mechanism, which is also explained in detail in Appendix A. In short, one could say that each layer of the graph neural network condenses information from the previous layers and uses it to compute new node features. This imposes an inherently local structure on the whole network, because only the information of connected nodes is available during each computation step. More information and the detailed architecture of our GNN is given in Appendix B.

For the training and testing procedure, we further divide the data into smaller batches of data. These batches contain a fixed number of input graphs which we call $batch_{size}$. In each training epoch, one batch after another is analysed and used to adapt the weights of the GNN accordingly. A training epoch is finished, when all batches have been completely processed. During the training it is important to be sure that all batches are balanced over different classes which might be present in the input. The batching of input data has the advantage that the weights are updated more often, which can be beneficial for faster and more refined learning results.

## Results

### Supervised learning of the state behaviour

**Fig. 1:**
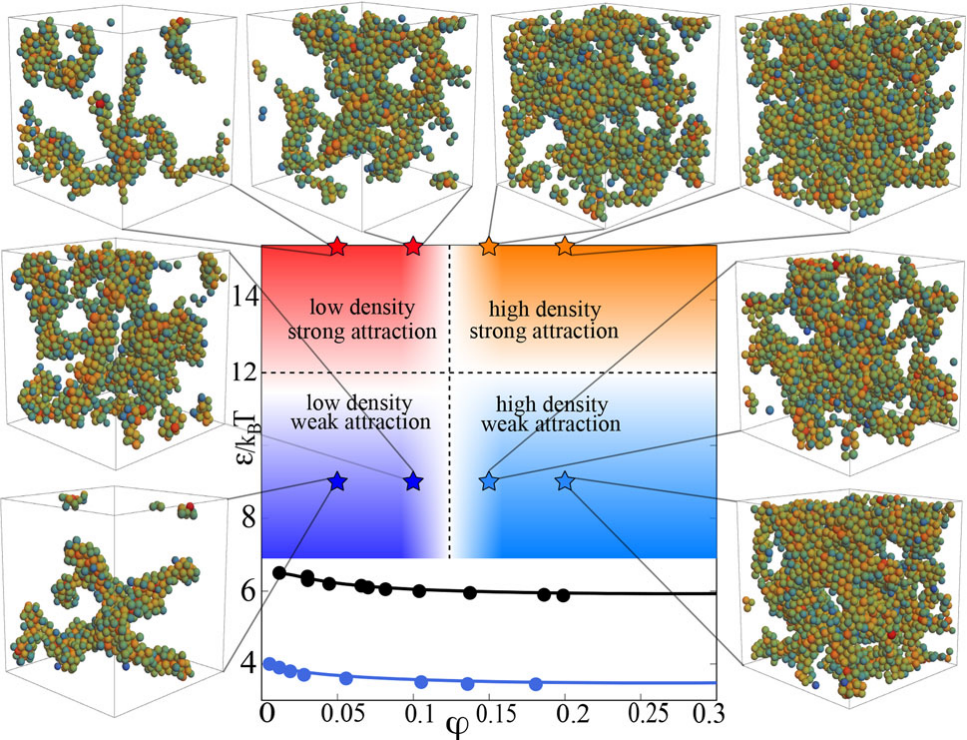
State diagram of the considered gel-forming system. Details of the model are given in Sect. 2. The blue and black lines represent the binodal line for inverse screening lengths $\kappa =\infty$ and $\kappa =15$, respectively. Coloured areas (red, orange, light blue and dark blue) stand for the 4 different classes of gel networks chosen for the article. Stars represent the parameter values used for training datasets, and the insets show exemplary configurations at the corresponding parameter values. Note that all the shown gel structures are metastable non-equilibrium structures. Binodal lines are obtained by explicit simulation of coexistence densities [19, 37]

As shown in our previous articles [19, 20] gel networks can be classified in a state diagram spanned by the magnitude of attraction $\epsilon$ and the packing fraction $\varphi$ (see Fig. 1). From visual observations it is hard to distinguish between the different structures, also the crossover in the packing fraction regime is hard to characterize. We show that it is possible to classify gel networks using supervised machine learning, meaning that we specify the corresponding class for each gel network before the training. In addition, it is also possible using unsupervised leaning as shown later in Sect. 3.2 where the GNN itself develops a suitable classification scheme.

#### Classifying gel networks according to the strength of attraction

For supervised classification tasks on the graph level, we use a 3-layer GNN. It consists of 3 GIN-blocks, a global mean pooling operation to get graph level predictions and an additional 3-layer perceptron to classify the obtained graph embedding. The hidden dimension $\mathrm{{dim}}_h$ is determined during training in the optimization process. The hidden dimension is the dimensionality of the neurons in the hidden layers of the GNN and therefore gives the dimensionality of the node embeddings during training. Other hyperparameters to be determined during the training process are the learning rate $\lambda$ and weight decay *w*. The learning rate is a measure of how fast the weights of the neural network adapt to new input. Very roughly speaking one could say the bigger the learning rate, the faster the network learns, but the less accurate the training will be. The weight decay parameter is used to prevent overfitting and leads to a slow change in time of the weights in the neural network. The GNN is not trained on whole gel networks, but only on connected subparts of it. The size of these subnetworks is given by their number of particles $N_{\text {sub}}$. This is done to increase the number of potential training samples, as from each simulated large gel network several smaller subnetworks can be extracted. For more details, we refer to Appendix section B and Fig. 9a–c.

**Fig. 2:**
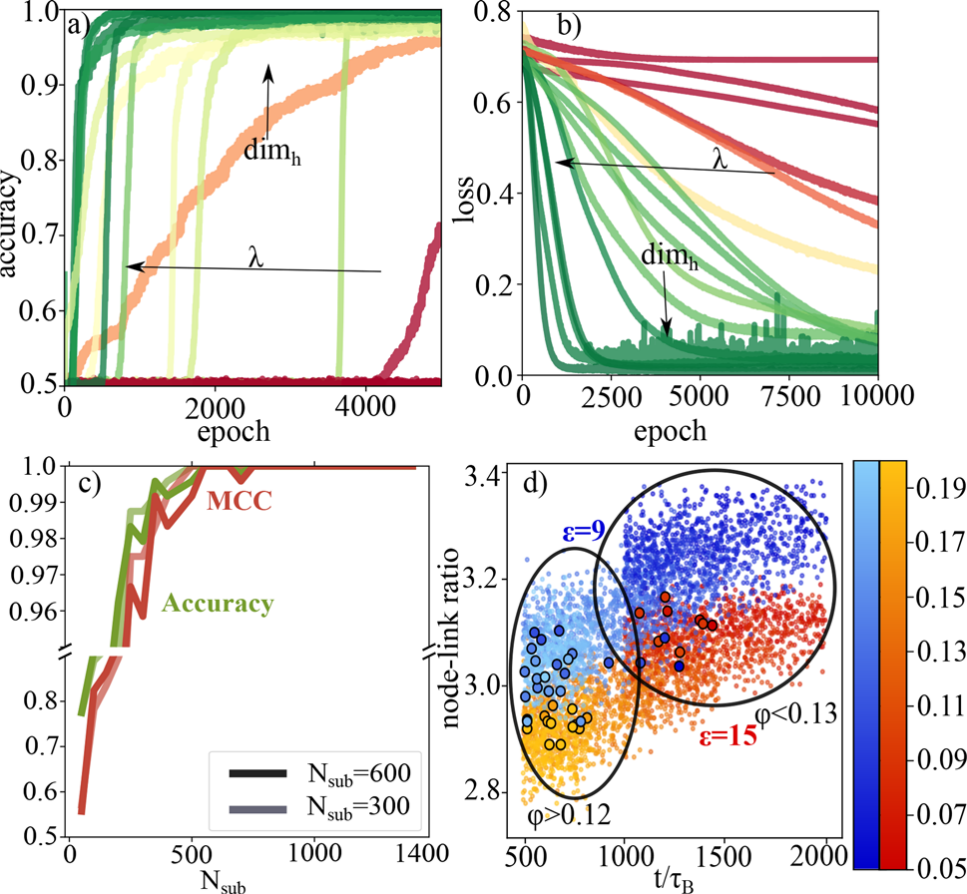
Results of supervised learning to classify according to attraction strength. **a** Accuracy of test dataset classification during training for different sets of hyperparameters. The data are continuously colourcoded from the lowest maximal accuracy value (reddish) to highest maximal accuracy value (greenish). **b** Loss function for the test dataset classification during training. The data are continuously colourcoded from the highest minimal loss value (reddish) to lowest minimal loss value (greenish). Together with **a** this is used to determine the optimal set of hyperparameters. **c** Extrapolation ability of the neural network trained on subgraphs of size 300 and size 600. **d** Location of classified samples (5000 networks with $N_{\text {sub}}=300$). Colours are chosen as in the state diagram in Fig. 1. Each circle stands for one gel network, small black circles denote misclassified samples, and the big black circles separate dense gels and dilute gel networks

Gel networks either belong to a weakly bound or a strongly bound regime, depending on the strength of the attraction between the particles. [19] Therefore, each training sample *i* is given a binary target value $y_i$ ($\epsilon =9 \rightarrow y_i=0$, $\epsilon =15 \rightarrow y_i=1$) to specify the regime to which it belongs. These binary targets are then used to train the GNN for binary classification whether an input gel network belongs to the weakly or strongly bound class (reddish or bluish part of the phase diagram in Fig. 1). The training procedure is conducted by first choosing a certain value for $\mathrm{{dim}}_h$ and *w*, then for these fixed values we test several $\lambda$-values to determine the value with the best accuracy and lowest loss. When for one value of $\mathrm{{dim}}_h$ a good value for $\lambda$ is found we choose a new value for $\mathrm{{dim}}_h$ and repeat the training. In the end, the pair of values is chosen with the lowest loss value and highest accuracy. $\lambda$ influences the training such that the GNN learns more slowly, but more accurately for lower $\lambda$-values, higher $\mathrm{{dim}}_h$ leads to more accurate training but makes the network susceptible for overfitting. Therefore, a good compromise of not too high values for $\mathrm{{dim}}_h$ and not too low values of $\lambda$ has to be found. In Fig. 2a, b, we plot the test accuracy during the training procedure to determine the optimal set of hyperparameters $\{\mathrm{{dim}}_{h}, \lambda , w\}$. The optimal set of hyperparameters for $N_{\text {sub}}=300$ was $\{32,0.00001, 0.001\}$ and for $N_{\text {sub}}=600$ we chose the same combination $\{32,0.00001, 0.001\}$ (see Table 1). We find that the GNN is able to classify the gel networks nearly perfectly concerning the attraction with only a very small amount of misclassified samples.

Also extrapolation to non-learned subgraph sizes is possible and shows good results (see Fig. 2c). To quantify this we use the accuracy and Matthews Correlation Coefficient [40] (MCC). Accuracy is the fraction of correct predictions divided by the total number of samples. It ranges between 0 and 1. Due to its easy interpretability it is well suited as a first estimate of the performance. This is true at least for balanced datasets as we use them here. On the other hand, the MCC is more stable against unbalanced datasets and has the advantage that for its calculation it takes into account true positive (TP), false positive (FP), true negative (TN) and false negative (FN) prediction values. This increases the informativity and reliability of the score.

$$\begin{aligned}&MCC\\&\quad =\frac{TP \cdot TN-FP \cdot FN}{\sqrt{(TP{+}FP)(TP{+}FN)(TN{+}FP)(TN{+}FN)}} \end{aligned}$$

Its values range between $-1$ and 1, with $-1$ being the worst value indicating perfect misclassification and 1 being the best value indicating perfect classification. The closer the MCC is to 1, the better the performance of the GNN. We compare the extrapolation ability of networks trained on subgraphs of size $N_{\text {sub}}=300$ and size $N_{\text {sub}}=600$ to understand the influence of the subgraph size on the extrapolation ability. Irrespectively of the chosen training subgraph size the network performance increases with subgraph size and shows excellent results for graphs with more than 400 nodes. Therefore, due to the differences in their local structure it is possible to train the GNN on small substructures and it will generalize well to bigger structures.

We further investigate the GNNs ability to distinguish which properties of the gel networks are used to classify the regime. In Fig. 2d, we show the link-node ratio of dilute and dense gel networks as a function of the simulation time. Misclassified samples are marked by small black circles. We find that most of the misclassified samples are located in the region where samples with $\epsilon =9$ and $\epsilon =15$ overlap. We call this region the link-node boundary. Therefore, this might be an indicator of an important quantity in the classification process. Our dataset preparation method makes it possible to distinguish as well in which density regime the misclassified samples are mainly located. For dilute gel networks ($\varphi \in [0.05,0.12]$), we use samples with simulation time $t>1000\tau _B$. For dense gels ($\varphi \in [0.13,0.20]$), we use $t>500\tau _B$. In both cases misclassification seems to be less likely for longer simulation times and there are more misclassifications in dense systems than in dilute ones.

#### Classifying gel networks according to the packing fraction

**Fig. 3:**
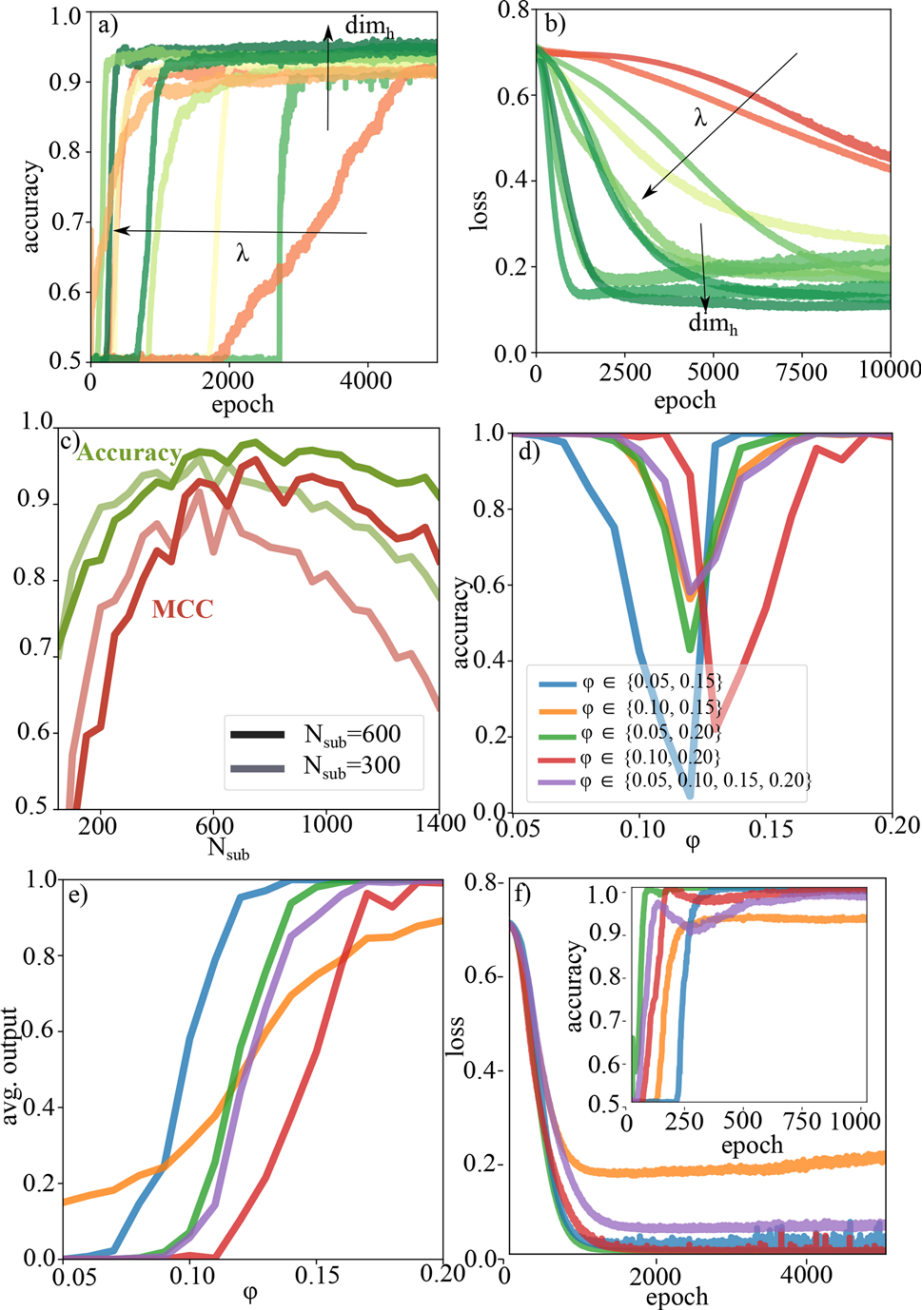
Supervised classification of packing fraction. **a** Test dataset accuracy for different hyperparameters. **b** Test dataset classification loss function used to determine optimal hyperparameters. Arrows show the qualitative influence of the hyperparameters. The colourcode in **a** and **b** is chosen as in Fig. 2**c** Extrapolation ability of the GNN trained on subgraphs of size 300 or 600. We evaluate 500 gels with densities $\varphi \in \{0.10,0.15,0.20\}$. **d** Classification accuracy for classifiers trained on different training sets. A manual threshold was set at $\varphi =0.12$. The best performance is seen for the classifier trained on all 4 densities. **e** Average output of GNNs trained on different combinations of packing fractions. The crossover density depends on the packing fraction combination in the training set. **f** Loss function and accuracy for the GNNs trained on different combinations of input packing fractions. $\varphi \in \{0.10,0.15\}$ shows slight overfitting

The next step of our analysis is the classification depending on the packing fraction of the gel networks. In recent work [20], we found that the dynamical and structural behaviour shows a smooth crossover at a packing fraction between $0.1 \le \varphi \le 0.15$. This crossover is not as pronounced as the overall changes when changing the attraction. Still we see that the neural network is able to classify gel networks according to structural differences that depend on the density. Note that the neural network does not know about the interaction potentials and the length scales therein. Therefore, the mean distance between particles does not tell anything about the packing fraction. Instead the network is trained on structural differences irrespective of length scales (that can be arbitrarily rescaled in the input without changing the results).

For the training, we construct binary target values for each training sample *i* according to $\varphi \le 0.12 \rightarrow y_i=0$ and $\varphi > 0.12 \rightarrow y_i=1$. In Fig. 3a, b we show the validation accuracy and loss during the training and optimization process. The GNN is again described by a set of 3 hyperparameters $\{\mathrm{{dim}}_h, \lambda , w\}$ as before. After training we find that the optimal set of hyperparameters for $N_{\text {sub}}=300$ is $\{16, 0.00001, 0.0001\}$ and for $N_{\text {sub}}=600$ is $\{32, 0.00001, 0.0001\}$.

Again the extrapolation ability to non-learned subgraph sizes works well as we show in Fig. 3c We compare networks trained on two different subgraph sizes ($N_{\text {sub}}=300$ and $N_{\text {sub}}=600$) as explained before. We find that the performance and the generalization ability is not as good as in the attraction case. The GNN trained on $N_{\text {sub}}=300$ generalizes not so well to larger graphs, as the one trained on $N_{\text {sub}}=600$. The performance of the GNN for $N_{\text {sub}}=300$ peaks at some distinct subgraph size and then decreases for larger graphs, while it decreases much slower for the classifier trained on $N_{\text {sub}}=600$. For smaller graphs both classifiers shows decreasing performance. In the last part of the supervised GNN analysis, we train 5 GNNs on different combinations of input packing fractions ($\varphi \in \{0.05, 0.20\}, \varphi \in \{0.05, 0.15\}, \varphi \in \{0.10, 0.15\}, \varphi \in \{0.10, 0.20\}$ and $\varphi \in \{0.05, 0.10, 0.15, 0.20\}$). We use subgraphs of size $N_{\text {sub}}=600$ for this. The aim is to see whether the crossover packing fraction from dilute to dense gel networks is located at the same value for all of the GNNs or if the value depends on the input packing fraction combinations during training. Therefore, we show in Fig. 3d the classification accuracy and in Fig. 3e the averaged GNN output for samples with packing fractions in the range $\varphi \in [0.05, 0.20]$. The averaged output can be seen as a measure of how certain the GNN is that the analysed samples belong to the dilute or dense regime. We find that as well for the accuracy as for the averaged output the crossover packing fraction depends on the chosen training combination of packing fractions. The averaged output shows sharp transitions except for the GNN only trained on $\varphi \in \{0.10,0.15\}$. In Fig. 3f, we show the test loss function and test accuracy of the 5 different GNNs during training. All of the GNNs show very good performance with nearly perfect accuracy except for the GNN only trained on $\varphi \in \{0.10,0.15\}$ which shows slight overfitting and lower accuracy values. Together with the behaviour of the averaged output, this can be interpreted as the consequence of structural similarities between dilute gel networks at $\varphi =0.10$ and dense gels at $\varphi =0.15$. These similarities complicate the GNN‘s task to extract properties which can be used to distinguish dilute and dense systems. In variance with that, for the other combinations of input packing fractions the structural differences are big enough to clearly distinguish the packing fraction regimes. Still the learned crossover packing fraction depends on the combination of input packing fractions during training.

**Fig. 4:**
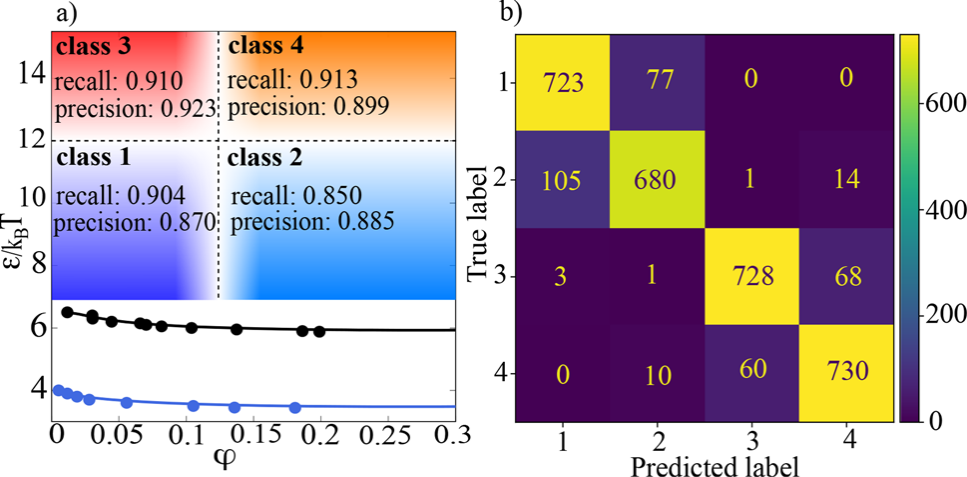
**a** Phase diagram with precision and recall scores of the combined classifier. Both values are close to 0.9 for all classes. **b** Confusion matrix of the combined classifier with 3200 samples (800 for each class) of size $N_{\text {sub}}=300$ taken from the evaluation datasets with $0.05 \le \varphi \le 0.2$ and $\epsilon \in \{9,15\}$

#### Simultaneous classification of attraction and packing fraction

We further combine the two single classifiers explained so far, to get a combined GNN. This GNN is able to classify data samples at the correct position in the phase diagram concerning the magnitude of attraction and the packing fraction at the same time. This means that we do not have a binary classification problem anymore, but 4 different classes

- Class 1: low density+weak attraction ($\varphi \le 0.12$, $\epsilon =9$)
- Class 2: high density+weak attraction ($\varphi >0.12$, $\epsilon =9$)
- Class 3: low density+strong attraction ($\varphi \le 0.12$, $\epsilon =15$)
- Class 4: high density+strong attraction ($\varphi >0.12$, $\epsilon =15$).

Therefore, the architecture of the GNN is slightly changed. The output and target value are now not single binary values, but a 2-dimensional vector with two binary entries determining packing fraction and attraction, respectively. We use the pretrained GNNs from before and determine the confusion matrix, as well as recall and precision of the single classes. Precision is for each single class defined as the ratio of true positive samples for each class divided by all predicted samples for the corresponding class, while recall is the ratio of true positive samples for each class compared to all samples which belong to the corresponding class. Therefore, precision can be interpreted as a measure of the quality of prediction and recall as a measure of sensitivity. Both values can be calculated using the confusion matrix, which stores the performance of the network classwise by displaying in each row the number of correctly predicted samples and for the misclassified samples also to which class they have been assigned. We find in Fig. 4a that both recall and precision show high values around 0.9. As to be expected from the performance of the single classifiers, the GNN is able to predict the attraction regime with higher accuracy than the density regime (see Fig. 4b). Nearly all misclassified samples are classified as members of the neighbouring density regime, not the neighbouring attraction regime. Meaning that mostly classes 1 and 2 are confused or, respectively, classes 3 and 4.

### Unsupervised learning of the state behaviour

To get more insight if an artificial intelligence is able to recognize small structural differences without prior preclassification we investigate unsupervised learning. In contrast to the supervised case, we now use a different architecture for the GNN. Details are given in Sect. B. This time the GNN consists of 5 GIN-blocks, followed by a global mean pooling operation and a 3-layer perceptron. The difference is now that the perceptron does not give one single value as output for each graph, but a vector of dimension $\mathrm{{dim}}_{lat}$. We call $\mathrm{{dim}}_{lat}$ the latent dimension, which will then be used for further classification using dimensional reduction algorithms. For this, we use principal component analysis (PCA) [41] or t-distributed stochastic neighbour embedding t-SNE [42]-analysis. In short terms the t-SNE searches for similarities in the very high-dimensional input data. Then, it compares this with a lower-dimensional probability distribution to get a low-dimensional embedding of the high-dimensional input. PCA-analysis is simpler in the sense that it tries to reduce dimensionality by translating, rotating and projecting the high-dimensional input in a low-dimensional output space. Mathematically speaking a principal axis transformation is applied. For more information we refer to the original publications [41, 42]. The desired output feature space was chosen to be 2-dimensional for both methods, as we expect attraction and packing fraction to be independent parameters.

For the unsupervised learning, the training procedure is different than before. The main difference is that no predefined targets exist. Therefore, it is necessary to construct targets, which in principal can change in every epoch, against which the loss is calculated. These are then used for updating the neural network in each learning epoch. The idea behind our target construction algorithm is that corresponding elements in the output vectors are calculated in the same way even for different samples. Therefore, different values of these elements in the output vectors can be used as an indication that the corresponding samples belong to different gel classes. As a consequence, one can quantify how much the different inputs change the predictions for the corresponding element and use this for target construction. In principle, we want to use a feedback that magnifies and classifies differences in the output vectors for different inputs.

**Fig. 5:**
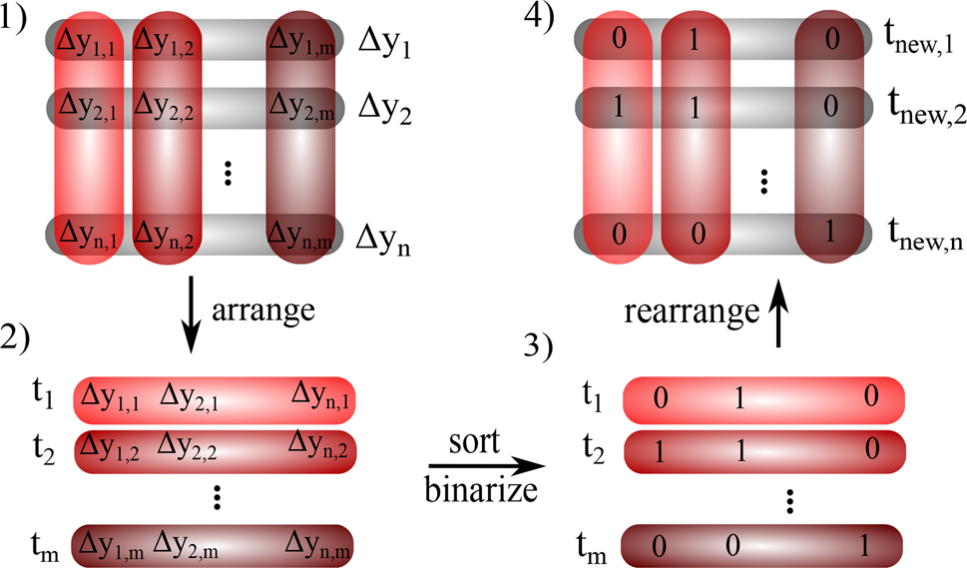
Algorithm for the target construction in unsupervised learning. 1 Calculate the difference between the previous output and the current output to get $\Delta y_i$ for all *i* in the batch. 2 Rearrange such that corresponding elements $\Delta y_{i,j}$ of all $\Delta y_i$ are arranged in a helper vector $t_j$. 3 Sort all $t_j$ in descending order and set the upper half to 1, the lower half to 0. 4 Invert the operation of step 2 and rearrange the elements of all $t_j$ in the original form. This rearranged form is then taken to calculate the new target vector by averaging with the prior target vector and normalizing the values to [0, 1]

We implement this idea in the following way: At first we initialize training and test datasets as for supervised training but without specifying target values. The model details are given in Appendix B. A neural network is used to generate an output vector of dimension $\mathrm{{dim}}_{lat}$ for each given input. We construct a target vector as explained in the following and depicted in Fig. 5. After the first epoch, the first output vectors are used to compute the first target vectors. We collect the values of the output vectors in each batch elementwise and arrange them into a new vector as shown in Fig. 5. We get $\mathrm{{dim}}_{lat}$ new vectors, each of length $batch_{size}$ such that we only compare corresponding elements of each output vector. These new vectors are then sorted in descending order. The first half of the elements in the ordered vector is assigned to 1, the second half is assigned to 0. Then, this is rearranged to the initial form of $\mathrm{{dim}}_{lat}$-dimensional vectors by inverting the previous arrangement. The corresponding vectors are then used as target vectors against which the loss is calculated and which are used for updating the GNN weights. The output vectors and the calculated target vectors are saved for the next epoch.

In all following epochs, the calculation is slightly different from the first epoch. Here we take the difference of the previously saved output vectors $y_{i,old}$ and the newly calculated predictions $y_{i,new}$ of the actual epoch. Then, the vectors $\Delta y_i=y_{i,new}-y_{i,old}$ are taken in each batch to perform the same ordering and binarization calculation as described above. The main difference now is that for the new target vector, we do not simply take the binarized vectors. We take the weighted average of the previous target vector and the newly calculated binary target vector to get continuous predictions. This vector is then normalized such that each element is in the range [0, 1]. The advantage of this method is that the target values cannot fluctuate so strongly as without averaging.

**Fig. 6:**
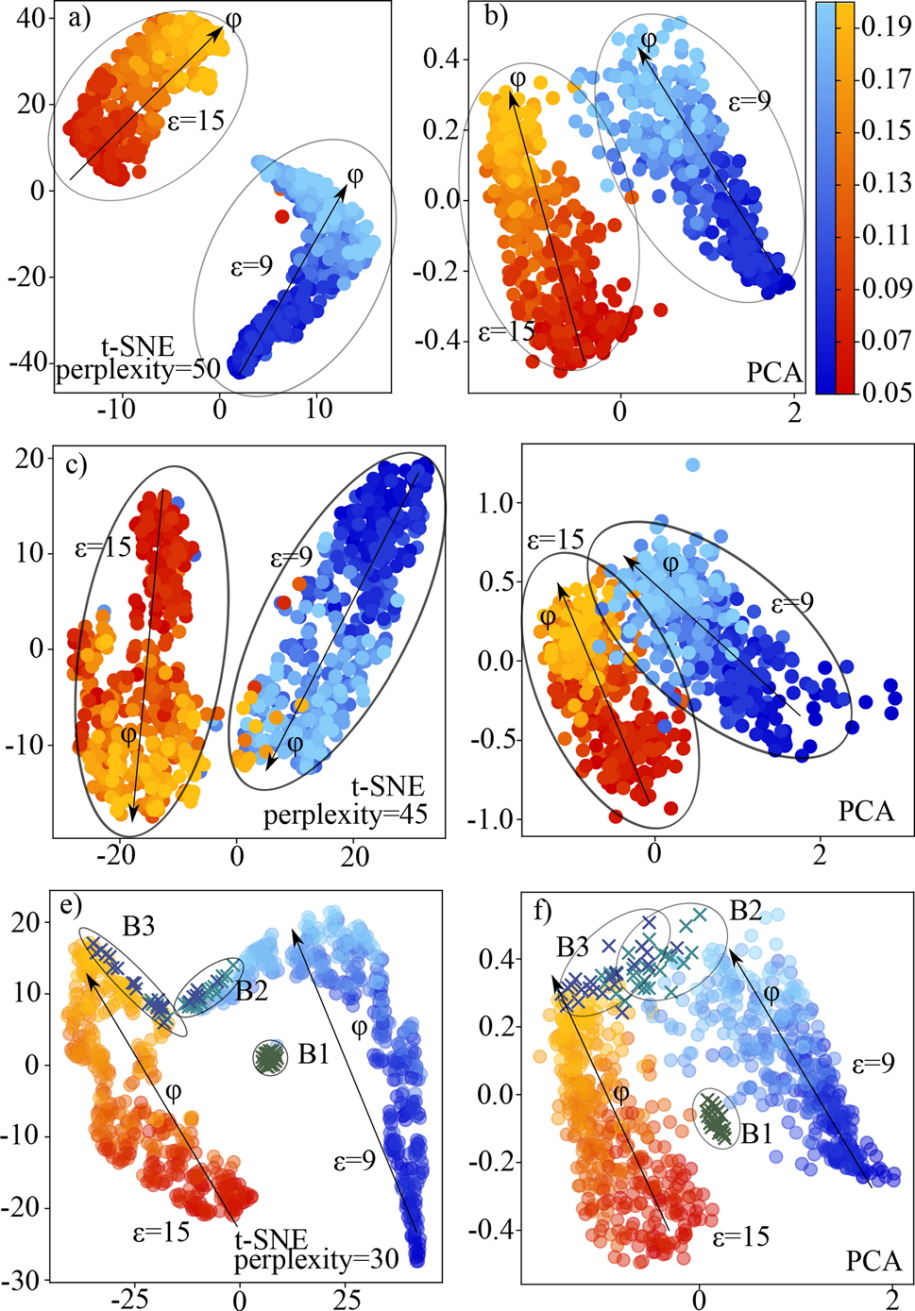
Unsupervised classification after dimensional reduction (Left column: t-SNE reduction, right column: PCA reduction). Bluish dots represent samples with $\epsilon =9$, reddish dots stand for $\epsilon =15$. Evaluation densities are colourcoded continuously from $\varphi =0.05$ dark colouring to $\varphi =0.20$ bright colours. **a**, **b** show results for a classifier trained on datasets of density $\varphi \in \{0.05,0.10,0.15,0.20\}$. We see for both reduction methods a clear separation of strongly ($\epsilon =15$) and weakly ($\epsilon =9$) connected gel networks and within the point clouds a clear continuous transition from dilute to dense gel networks. **c**, **d** are trained on data samples with densities $\varphi \in \{0.10,0.11,0.12,0.13,0.14,0.15\}$ and thus show the extrapolation of the classification to non-trained densities (higher and lower densities) in the evaluation dataset. **e**, **f** show the same as **a** and **b**, but with experimental data points included. Crosses labelled B1, B2, and B3 correspond to experimental data from [18]

For the analysis of the unsupervised GNNs we use in total 500 subgraphs of size $N_{\text {sub}}=600$ taken from both attraction regimes ($\epsilon =9$, $\epsilon =15$) and continuous packing fraction range $\varphi \in [0.05,0.20]$. The chosen latent dimension of the output vector is $\mathrm{{dim}}_{lat}=100$. We find that the network is indeed able to separate the gel samples corresponding to different classes. In Fig. 6a, b, we show that as well in the PCA as in the t-SNE reduction algorithm two distinct sets are formed. Samples which are part of the strongly connected networks are depicted by reddish points, while weakly connected networks are depicted using bluish points. The density is colourcoded in the range $0.05 \le \varphi \le 0.20$ from dark to bright blue or red. The separation in the attraction regime is very clear and seems to be easier for the network. This is in agreement with our findings from supervised learning where the attraction classifier outperformed the density classifier. In the density regime we see in both subsets a continuous transition from dilute networks to dense networks, indicating that also here the unsupervised network is able to group similar density networks and distinguish them from other densities. This shows the interpolation ability of the GNN, as it was trained only on a subset of the analysed densities. What we do not see is a clear transition for any intermediate density. If such a transition exists, it probably only can be detected by also taking dynamic properties into account that differ between small and large densities as shown in [20].

We compare GNNs trained on densities $\varphi \in \{0.05, 0.10,0.15,0.20\}$ with GNNs trained only on the intermediate densities $\varphi \in \{0.10,0.11,0.12,0.13,0.14,0.15\}$ to see whether the unsupervised network is able to not only interpolate, but also correctly extrapolate non-learned densities. In Fig. 6 c, d, we see that for PCA and t-SNE the classification results are not as clear as for the interpolation case shown before. Still we see for both cases two distinct clusters for the attraction regime and in these clusters a continuous crossover from dilute to dense gel networks as before. For high densities data for the two attraction strengths seem to slightly overlap in the PCA case shown in Fig. 6d. Using t-SNE analysis, the classification is clearer with two distinct clusters for the two different attraction strengths and no overlap any more. Furthermore, the continuous classification of density appears again. Therefore, we conclude that the network is able to successfully classify and extrapolate to non-learned densities. Indeed these results suggest that there are structural differences depending on density and attraction which the neural network is able to find. It may be insightful to understand what exactly the GNN is using to classify the networks. This indeed is a challenging task, as the latent feature space which is used for classification is very high dimensional.

In Fig. 6e, f, we apply the GNN that has been trained with simulated data with the goal to classify experimental data. For our analysis we used 16 subgraphs of size $N_{\text {sub}}=600$ from each considered point in the phase diagram of [18]. For details of the experimental procedure used to obtain the data we refer to the original publication.[18] The data samples B1, B2 and B3 all have approximately the same packing fraction (see state diagram in Fig. 2 in [18] for details). Note that the packing fraction in [18] is defined differently from the packing fraction here as a different interaction potential and thus a different radius of the particles is considered. The effective packing fraction is affected by the salt concentration [43]. In the experiments, the salt concentration and therefore the repulsion between the particles was changed. As a touching criterion used in the graph construction we use $r_{ij}\le 2.2\mu m$, which roughly corresponds to the location of the first minimum in *g*(*r*). For details see sect. 2.2. Sample B1 is taken in an unpercolated state. It is not a homogeneous fluid, but already contains clusters. B2 is taken between the lines of continuous and directed percolation and B3 is a percolated gel network that is out of equilibrium. The percolation in the system of the experiment occurs in the phase-separated heterogeneous regime [43]. Even if the interaction potential is different from the one used in our simulations, we still see that the GNN is able to position the experimental data samples at meaningful positions. For the percolated case B3, we see that it is located close to the high density samples of strongly bound networks from the simulations which indeed are the corresponding percolated clumpy gel networks [20]. Going along the experimental path from B3 via B2 to B1, according to the classification of the GNN the systems correspond to gels at lower packing fraction. This corresponds to the direction towards unpercolated systems [20]. We see that B2 is located closer to the weakly bound states and B1 is classified in between the two clusters, thus indicating a different network state then the learned states. Indeed, unpercolated systems have not been trained; therefore, it makes sense to locate B1 at a position separated from the two clusters. The separation between B3 and B2 is more pronounced when we use t-SNE analysis. In PCA, both show a significant overlap, but the general trend is still visible.

Note that locating experimental data in the state diagram is non-trivial [18, 43] as it is hard to determine the strength of attraction or even the exact effective radius in an experiment. Furthermore, any mapping on a theoretical or simulation model might depend on details of the considered interaction potential. Therefore, the unsupervised learning method might provide an additional clue to which gel network an experimental system structurally is close to, even though a different interaction potential might be used to train the employed GNN. It will be important for future work to find out to what extent the similarly classified gels are really similar concerning physical properties. Here, we have at least shown that the trend along a specific path can be classified correctly.

### Reconstruction of gel network skeleton structures

**Fig. 7:**
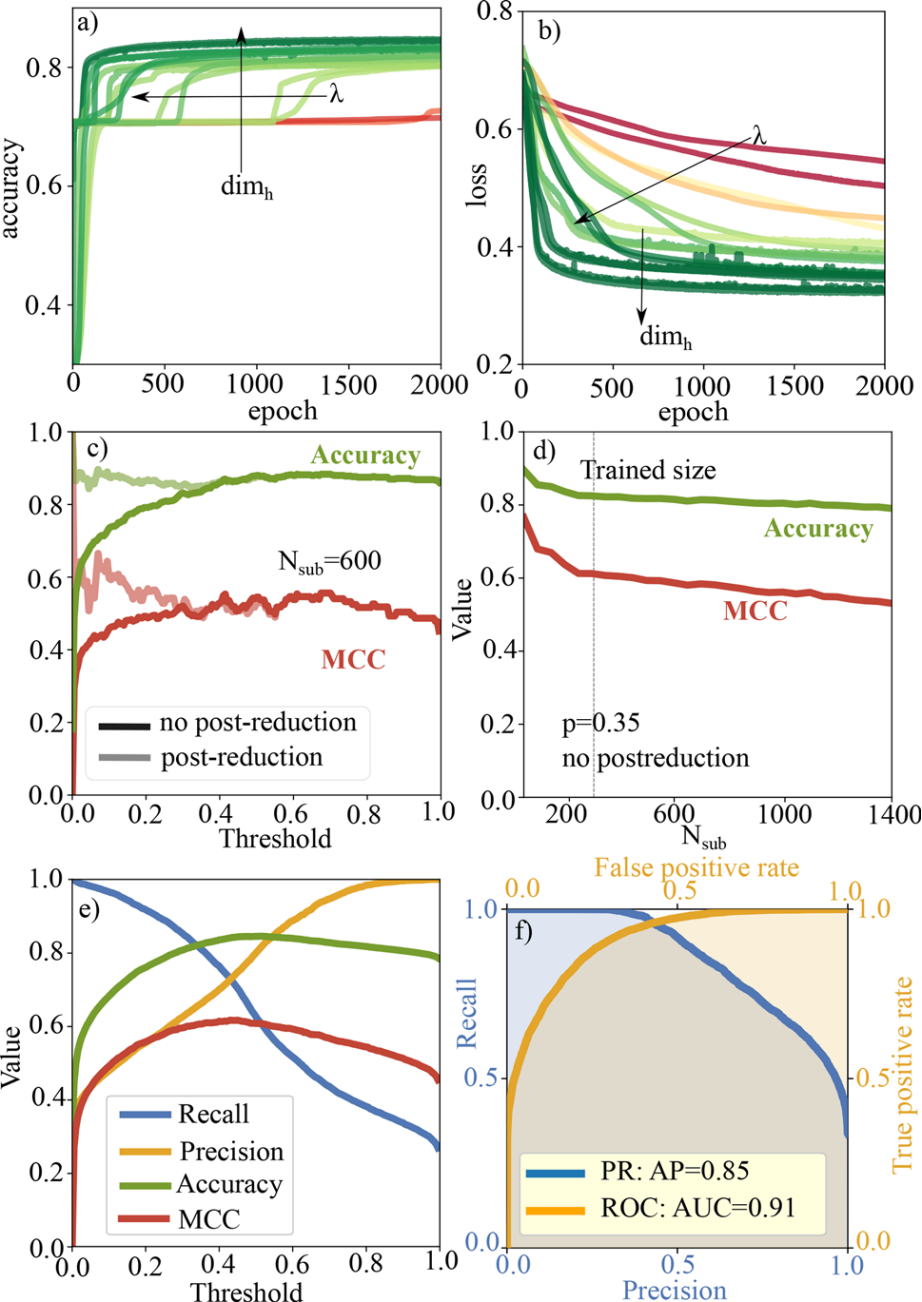
Performance of network reduction with GNNs. **a**, **b** Test accuracy and test loss during training for different hyperparameters. The colourcode is the same as in Fig. 2**c** Accuracy and MCC of the chosen GNN for graphs of size $N_{\text {sub}}=600$ with additional analytic postreduction and without. Choosing a small classification threshold with additional postreduction increases performance of the network and decreases computation time. **d** Extrapolation ability to subgraphs of different sizes. The GNN is trained on graphs of size $N_{\text {sub}}=300$ and shows good generalization to bigger gel networks. **e** Performance metrics for the chosen hyperparameters as a function of classification threshold *p*. **f** Precision-Recall curve (blue) and Receiver operating characteristic ROC (orange) for the chosen hyperparameters

In this section, we test the node classification ability of GNNs by constructing backbones from simulated gel networks.[19] Reduced networks are minimal connecting structures, which are determined by neglecting all particles that are not crucial for the connectivity of the whole network. In principle one could also thin out gel structures by numerical approaches based on binarizing a raster image of it. However, the advantage of thinning out a network as a graph is that the resulting skeleton network is still on a particle level. This is because the graph is based on particles as nodes and connections as links. Unfortunately, the calculation is computationally expensive and scales at least with $N^2$, where *N* is the particle number.

Therefore, the idea is to train a neural network which can be used to skeletonize simulated particle networks. For each node, the GNN determines whether the corresponding particle is in the reduced network or not. The target structures that are used for training are the reduced networks determined as in [19]. As shown in more detail in Sect. B, we use a 5-layer GNN followed by a 3-layer perceptron for classification. This time the pooling layer we use in the previous sections is missing, because we do not want to get graph level predictions, but node level classification. Therefore, the learned node embeddings after the GNN are directly given to the classification perceptron. Hyperparameters to determine during the training process are again $\{\lambda , w, \mathrm{{dim}}_h\}$. In Fig. 7a, b, we show the accuracy and loss curves of the test data obtained during the training of the neural network to determine the optimal set of hyperparameters. Accuracy curves either start at around 0.7 or show a plateau at this value. This is because during the analytic construction of the skeletonized network approximately $70\%$ of the particles are neglected. Therefore, the dataset is unbalanced with $\approx 30\%$ positive samples and $\approx 70\%$ negative samples. So if the network just learns to neglect all particles, the accuracy value is 0.7. Therefore, it is important to check that the network does not get stuck in this state, but learns to distinguish between the two types of nodes to reach higher accuracy values.

The output of the neural network is a $N_{\text {sub}}$-dimensional vector with continuous values between 0 and 1. To decide whether a node belongs to the reduced network or not, it is necessary to choose a classification threshold *p* which is used for binarization of the output vector. If the predicted value is higher than *p* the corresponding node is assigned to the skeleton, if the value is smaller than *p* the node is neglected. In Fig. 7e we show recall, precision, accuracy and MCC as defined before for the chosen optimal hyperparameter set $\{64, 0.0001, 0.001\}$ as a function of classification threshold. We see that the performance of the network depends on the classification threshold and that the best performance is obtained for values $p\approx 0.35-0.4$ as the metrics here show their maximum. In Fig. 7f, we show the precision-recall curve and the Receiver operating characteristic ROC for this classifier at $p=0.35$. The ROC compares the true positive rate (TPR) of the classifier with its false positive rate (FPR). Random classification would result in a diagonal line in the plot, the more the curve deviates from this diagonal, the better the classification. For a perfect classifier TPR would be 1 and FPR would be 0, resulting in the point (0,1) in the ROC-space. A classifier which assigns each sample to the positive class in contrast would be located at (1,1). A measure for this is the area under the curve (AUC), where values near 0.5 stand for random classification, while 1 would indicate perfect classification. The precision-recall curve has a similar interpretation. It compares the recall of the classifier with the corresponding precision. A random classification would result in a horizontal line, meaning that for each precision recall is the same. Likewise as for the ROC the area under the curve, this time called average precision is used to evaluate the performance. Again values near 1 indicate very good performance, while smaller values stand for worse performance. We obtain good values for the average precision and the area under the curve indicating good performance of the GNN. Concerning the extrapolation ability of the network we show in Fig. 7d (for $p=0.35$) that it slightly decreases for networks larger than the trained network size. Still the GNN shows good performance even for networks which are nearly 5 times as big as the trained networks.

**Fig. 8:**
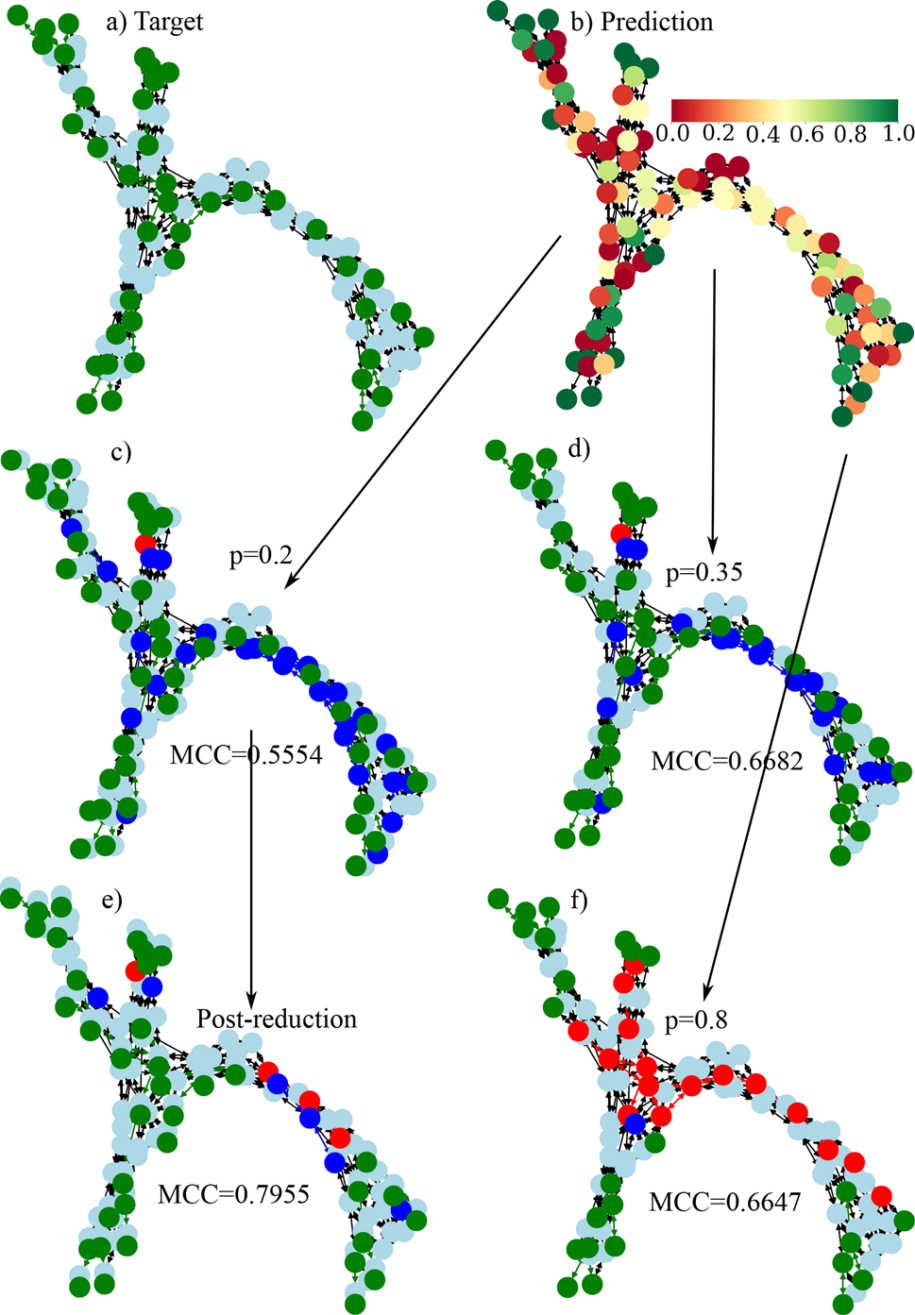
Determination of the reduced network of a subgraph with 100 nodes depending on the chosen threshold value. **a** shows the ideal output after the analytic reduction algorithm. **b** shows the predictions of the neural network, colours stand for different probabilities of a node belonging to the reduced network or not. **c**, **d** and **f** show reduced networks for threshold values of $p=0.2$, $p=0.35$ and $p=0.8$, respectively. Green nodes are correctly classified (true positive), red nodes would belong to the reduced network but are not reconstructed correctly (false negative) and dark blue nodes are labelled positive, but do not belong to the original reduced network (false positive). We notice that for too high threshold values as in f) the network does not perform good. For small threshold *p*=0.2 nearly all of the original nodes are reproduced, but many non-necessary nodes are also labelled positive. Nevertheless a reduction of the total node number is achieved and the output network can be used for further analytic reduction. **e** shows the analytic postreduced network after prereduction with *p*=0.2

For optimal performance, it is important to keep in mind how the classification threshold influences the final result. Sometimes it may not be the best choice to use $p=0.35$ even if the metrics show their maximum here, because it may still happen too often that crucial nodes are neglected. Then, it may be beneficial to use even lower threshold like $p=0.2$. The consequence shown in Fig. 8 is that nodes, which should not be part of the skeleton, are still present in the output network. But the advantage is that only a very small number of crucial nodes may be neglected mistakenly. This method still leads to massive reduction of the network size and gives the opportunity to postanalyse the prereduced network using the standard method of Ref. [19]. This will then converge faster as the number of particles already has already been reduced. In Fig. 7c, we show for subgraphs of size $N_{\text {sub}}=600$ that accuracy and MCC increase for additional postreduction if initially the classification threshold is too low. For high classification thresholds, this postanalysis does not change the result as no false-positive nodes appear. We conclude that it is beneficial to combine machine learning prereduction with conventional postreduction to get the best results.

The advantage of this approach is that once the network has been trained it runs with time complexity $\mathcal {O}(N)$ where *N* is the number of particles in the network, because we only use local node features which are calculated in linear time. Therefore, the computationally expensive analytic calculation of the conventional method can be accelerated by combining it with a prereduction using GNNs, such that the number of nodes to be analysed with the conventional method has already been reduced.

## Conclusions

Our machine learning approach using graph neural networks reveals that it is possible to classify gel networks depending on their packing fraction and the interparticle attraction both by using supervised or unsupervised learning. In the supervised learning approach, we found excellent performance for the discrimination of different attraction regimes and very good performance for the density regimes. Due to the local structure of message passing graph neural networks we expect the GNN to be able to well generalize the learned properties to larger graphs and we find that extrapolation is possible for both classifiers. We have not observed any sharp transition upon changing the density. To locate the structural crossover that occurs between $0.10 \le \varphi \le 0.15$ [20] seems to be hard. However, networks with smaller or larger packing fraction are classified in a reliable way indicating that there are indeed significant structural differences between dilute and dense gel networks.

Interestingly, even unsupervised learning shows that it is possible for the GNN to detect such structural differences when they were not told about possible differences before. Furthermore, the classification obtained by unsupervised learning can be mapped on the classification scheme used in supervised learning that is based on the state diagram. The classification concerning attraction is very sharp, as to be expected from the supervised approach. Distinguishing according to packing fraction seems to be more difficult, but still a broad spread of the output for different densities is visible in the resulting data. Interpolation to non-learned densities shows a good performance, while extrapolation does not perform as well because then the gels used during training probably were too similar. These results emphasize again that there seems to be no sharp structural transition between dilute and dense gels. However, even if the structural differences between dilute gel networks and clumpy gels are not obvious to the bare eye, they seem to be large enough to distinguish these structures. This finding leads to the question what the structural features are that the GNNs use to differentiate the gel networks. Identifying and characterizing possible characteristic structural differences is an important goal for future research. This may be helpful to characterize experimental gel structures, which a priori is not an easy task, as the precise interaction potential often is unknown.

Computation of reduced networks using an analytic approach is a computationally expensive task. We show that it is possible using machine learning and node classification to reconstruct reduced networks. It is important to keep in mind that the classification threshold strongly influences the performance. We argue that it is usually better to choose this threshold below instead of at or above the value where the most accurate output is expected. This leads to misclassifying in the sense that false-positive classifications occur more often and false-negative are suppressed. However, as the number of particles has been reduced substantially, the false-positive results can then be further reduced using the conventional procedure. In contrast, setting the threshold too high would lead to too many false-negative results and it would not be possible anymore to reconstruct the skeleton structure.

Using our significantly faster method for backbones determination based on GNNs, even the analysis of the backbone dynamics over longer time scales becomes accessible for future studies.

In summary, we have shown that it is possible to use message passing graph neural networks to analyse simulated gel network data. The results even give some insight into where significant structural differences between gels occur. Furthermore, we have demonstrated that even for experimental data the GNNs can correctly describe the trend of where the system should be located in comparison with the simulations that were based on different, simplified interaction potentials. For future research, it would be important to understand what the classification of the GNNs means concerning the physics of the gels like their rheological or mechanical properties. Furthermore, it might be interesting to find out how this approach generalizes to other interaction potentials and even more importantly what are suitable simplified pair interactions that can be used for training to classify systems with even more complex interactions.

## Figures and Tables

**Fig. 9:**
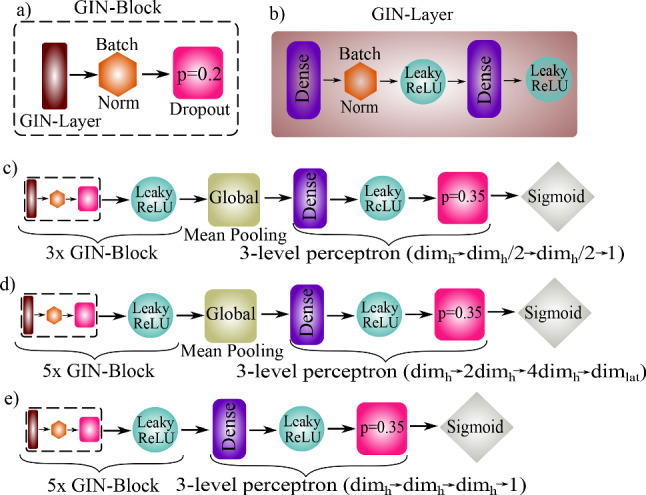
Architecture of the employed GIN used to learn node embeddings for each node. The GIN layer itself consists of dense (linear) layers, batch normalization layers [44] and two nonlinear LeakyReLU-activation functions. This type of layer together with another batch normalization and a dropout layer forms a GIN-block which is repeated *k* times. The further treatment of the result after the *k* GIN-blocks depends on the classification problem. For graph level classification, a global mean pooling layer is stacked with a conventional multi-layer perceptron, while for node level classification the pooling layer is omitted and the result is directly given to the conventional classifier

## Data Availability

The data that support the findings of this study are available from the corresponding author upon reasonable request.

## Node features and data preparation

For each node in the graph, a set of 6 distinct abstract graph level node features is computed using [39]:

- Node degree
- Number of edges in ego net of radius 1
- Number of edges in ego net of radius 2
- Average node degree of adjacent nodes
- Clustering coefficient
- Degree centrality

Using message passing graph neural networks has the advantage that they are not limited by the size of the training graphs, as only the local environment of each node is used directly in the training process they can be trained on small samples and then be applied to much bigger graphs. We take advantage of this property by training GNNs on small connected substructures of size $N_{\text {sub}}$. These are extracted using breadth first search on the graphs until the desired number of nodes has been chosen. Breadth first search is chosen, because it ensures in contrast to depth first search that the whole neighbourhood of each node is part of the subgraph, as on its way through the graph it first considers the whole neighbourhood of a node before considering the next node.

We used training sets of size 2048 distributed in 16 batches of size $batch_{size}=128$ and test data sets of size 512 in 4 batches of size $batch_{size}=128$ for supervised learning of graph classification and reduced network construction. The used subgraph size was either $N_{\text {sub}}=300$ or $N_{\text {sub}}=600$ if not stated otherwise in the text and as shown in Table 1. For unsupervised learning of graph classification, we used training sets of size 1024 distributed in 32 batches of size $batch_{size}=32$ and test data sets of size 256 in 8 batches of size $batch_{size}=32$. Here the used subgraph size was $N_{\text {sub}}=600$. We made sure that our dataset is balanced in the sense that from each simulated dataset and from each of the 4 regions in the phase diagram the same amount of samples is present in the training and test dataset.

**Table 1:** Table of optimal hyperparameter values determined in the training process for the different GNNs used in the article.

Section learning type	Classification level regime	$N_{\text {sub}}$	$\lambda$	*w*	$\mathrm{{dim}}_h$	$\mathrm{{dim}}_{lat}$	Batch size	Training samples Training set
3.1.1 supervised	Graph level attraction	300	$10^{-5}$	$10^{-3}$	32	1	128	2048 $\varphi \in \{0.05,0.1,0.15,0.2\}$ $\epsilon =9, \epsilon =15$
3.1.1 supervised	Graph level attraction	600	$10^{-5}$	$10^{-3}$	32	1	128	2048 $\varphi \in \{0.05,0.1,0.15,0.2\}$ $\epsilon =9, \epsilon =15$
3.1.2 supervised	Graph level packing fraction	300	$10^{-5}$	$10^{-4}$	16	1	128	2048 $\varphi \in \{0.05,0.1,0.15,0.2\}$ $\epsilon =9, \epsilon =15$
3.1.2 supervised	Graph level packing fraction	600	$10^{-5}$	$10^{-4}$	32	1	128	2048 $\varphi \in \{0.05,0.1,0.15,0.2\}$ $\epsilon =9, \epsilon =15$
3.1.2 supervised	Graph level packing fraction	600	$10^{-5}$	$10^{-4}$	32	1	128	2048 $\varphi \in \{0.1,0.15\}$ $\epsilon =9, \epsilon =15$
3.1.2 supervised	Graph level packing fraction	600	$10^{-5}$	$10^{-4}$	32	1	128	2048 $\varphi \in \{0.05,0.15\}$ $\epsilon =9, \epsilon =15$
3.1.2 supervised	Graph level packing fraction	600	$10^{-5}$	$10^{-4}$	32	1	128	2048 $\varphi \in \{0.1,0.2\}$ $\epsilon =9, \epsilon =15$
3.1.2 supervised	Graph level packing fraction	600	$10^{-5}$	$10^{-4}$	32	1	128	2048 $\varphi \in \{0.05,0.2\}$ $\epsilon =9, \epsilon =15$
3.2 unsupervised	Graph level both	600	$10^{-4}$	$10^{-4}$	64	100	32	1024 $\varphi \in \{0.05,0.1,0.15,0.2\}$ $\epsilon =9, \epsilon =15$
3.2 unsupervised	Graph level both	600	$10^{-5}$	$10^{-5}$	64	100	32	1024 $\varphi \in [0.1,0.15]$ $\epsilon =9, \epsilon =15$
3.3 supervised	Node level	300	$10^{-4}$	$10^{-3}$	64	$N_{\text {sub}}$	128	2048 $\varphi \in \{0.05,0.1,0.15,0.2\}$ $\epsilon =9, \epsilon =15$

The given hyperparameters were used throughout the paper when the corresponding GNN was used

## Graph neural networks and GNN architecture

Graph neural networks are used as we want to determine node features on a graph that represents the gel network. We assign 6 distinct graph-level node features to each node (see above) and do not use edge features. The used node features are shown in 2.2. We use graph isomorphism networks (GIN)[34], which are a special sort of message passing neural networks [35]. That means that in each layer the information from the previous layer is used to compute new node embeddings for each node. The GIN architecture is shown in Fig. 9b and implemented using the built-in method GIN*Conv* from pyTorch geometric [45]. To compute the new node embedding for each node *i* a two-step algorithm is used. First the node embeddings of *i* and all its neighbours (directly connected nodes) are summed, then this result is given to a multi-layer perceptron(MLP). In our case the MLP consists of two dense layers with hidden output dimension $\mathrm{{dim}}_h$, with batch normalization and LeakyReLU activation functions in between. The LeakyReLU functions introduce nonlinearity in between the layers, which is needed to train the network and batch normalization layers have been shown to effectively accelerate training of deep neural networks [44]. The single GIN-layers are combined to GIN-blocks (Fig. 9a) using an additional batch normalization [44] and dropout layer [46] ($p=0.2$) for regularization. Then, depending on the classification tasks several of these GIN-blocks are stacked as shown in Fig. 9c–e. This message passing process shows the inherently local structure of graph convolutional networks, as in each layer only local neighbourhood properties are used. Therefore, the number of stacked GIN-layers can be seen as an approximation the range of information which is used for the computation of node embeddings. In the case of global graph classification, we stack 3 GIN-blocks, meaning that the features of third neighbours are the furthest information which is available to each node.

As loss function for all supervised models, we use binary cross-entropy, as this is well suited for binary classification tasks.

$$\begin{aligned} L_{\text {BCE}}=\frac{1}{N}\sum y_n \text {log}(x_n)+(1-y_n) \text {log}(1-x_n) \end{aligned}$$

For the unsupervised learning, we use MSELoss from pyTorch.

$$\begin{aligned} L_{\text {MSE}}=\frac{1}{N}\sum (x_n -y_n)^2 \end{aligned}$$

where $x_n$ are predicted values from the neural network and $y_n$ are the corresponding target values. We use an RAdam-optimizer[47] with learning rates $\lambda$ and weight decay *w* which are hyperparameters determined in the optimization process.

### Phase diagram classification

For classification on the graph level, we take the individual node embeddings and construct a graph level embedding by taking the mean of all the learned node embeddings. Other possibilities would be to sum over the node embeddings or to concatenate them. The averaged node embedding vector thus has the size $\mathrm{{dim}}_h$ and is then given to a fully connected classifier, consisting of a 3-layer combination of dense layers, dropout layers and LeakyReLU activations in between. The size of the outputs hidden dimension after the dense layers depends on whether we use supervised or unsupervised learning. As shown in Fig. 9c, d for supervised learning the first dense layer has output size $\frac{\mathrm{{dim}}_h}{2}$ and the last output has size 1, while for unsupervised learning the output size increases to multiples of $\mathrm{{dim}}_h$ and the output size of the last layers is another hyperparameter $\mathrm{{dim}}_{lat}$. The last activation function is a sigmoid function, such that the output of the network is between 0 and 1, thus indicating a binary label to which class the network belongs.

### Reduced network reconstruction

For classification on the node level, i.e. the reconstruction of reduced networks, each single node embedding after 5 layers of GIN-blocks is given separately to a three layer fully connected classifier which outputs a continuous value between 0 and 1 indicating the probability whether a node *i* belongs to the reduced network or not. In between the classifiers layers we use a combination of LeakyReLU-activation-functions and dropout layers with dropout probability $p=0.35$ to introduce nonlinearity and for regularization. After the last layer we use a sigmoid function to get probability values between 0 and 1.

To summarize the network consists of stacked GIN-blocks which compute node embeddings by using only local information from the neighbourhoods of the nodes. These node embeddings are then either further compressed by global mean pooling or directly given to multi-layer classifiers. The hyperparameters to optimize during training are the learning rate $\lambda$, the weight decay *w*, the hidden dimension of the dense layers $\mathrm{{dim}}_h$ and for unsupervised learning the size of the output vector $\mathrm{{dim}}_{lat}$. The whole GNN is implemented using pyTorch [48] and pyTorch geometric [45] and evaluated using NumPy [49], networkX [39] and Matplotlib [50].
